# A Review of Pirfenidone as an Anti-Fibrotic in Idiopathic Pulmonary Fibrosis and Its Probable Role in Other Diseases

**DOI:** 10.7759/cureus.12482

**Published:** 2021-01-04

**Authors:** Parth V Shah, Prachi Balani, Angel R Lopez, Chelsea Mae N Nobleza, Mariah Siddiqui, Safeera Khan

**Affiliations:** 1 Medicine, California Institute of Behavioral Neurosciences & Psychology, Fairfield, USA; 2 Internal Medicine, California Institute of Behavioral Neurosciences & Psychology, Fairfield, USA; 3 Psychiatry, California Institute of Behavioral Neurosciences & Psychology, Fairfield, USA; 4 Neurology, California Institute of Behavioral Neurosciences & Psychology, Fairfield, USA; 5 Neurology, St. George's University, True Blue, GRD

**Keywords:** pirfenidone

## Abstract

Fibrosis is the result of chronic inflammation and is known to pathologically occur in many organs and systems. Pirfenidone (PFD) is an anti-fibrotic known for its use in idiopathic pulmonary fibrosis (IPF). In addition to being an anti-fibrotic, it acts as an anti-inflammatory and antioxidant as well. There have been studies on PFD in other diseases, some clinical and others preclinical. We have compiled and reviewed them to highlight just how widespread PFD use could be. Among many benefits of PFD in IPF, PFD has effectively improved patients' survival in those who had an acute exacerbation of IPF and has reduced respiratory-related hospitalization, among few others. PFD also has shown an improvement in vital capacity in patients with chronic hypersensitive pneumonitis. Also, it has demonstrated anti-fibrotic effects in systemic sclerosis-associated interstitial lung disease. In other diseases outside the lungs, PFD has reversed insulin resistance and proven to be effective in non-alcoholic steatohepatitis (NASH). It has prevented blindness post-alkali injury to the eye and has proven to decrease the proliferation of mesothelioma cells, just to name a few. This review encourages further research in connection with PFD and its use in other diseases and PFD pros in IPF.

## Introduction and background

Fibrosis is scarring tissue and occurs when excess extracellular matrix material is replaced by collagen and fibronectin in response to cellular damage. Fibroblasts are the producers of a variety of substances found in the extracellular matrix. When these cells transform via a range of signaling programs, they differentiate into many types of cells. Myofibroblasts are one of its major phenotypes. They play a significant role in excessively synthesizing and secreting the extracellular matrix. They also hold resistance against apoptosis. These cells are susceptible to chemokines, cytokines, and growth factors [1]. These cells can also be generated from other sources and activated by multiple mechanisms, playing an essential role in fibrosis [2]. Any chronic inflammatory state, such as idiopathic pulmonary fibrosis (IPF), rheumatoid arthritis, scleroderma, end-stage renal disease, and myocardial infarction, can lead to fibrosis [3].

Pirfenidone (PFD) is an anti-fibrotic drug known to act on multiple fibrogenic pathways to reduce fibrosis in IPF of the lungs. It downregulates the production of growth factors [4]. It also decreases fibroblast proliferation and influences transforming growth factor-beta (TGF-β)-mediated differentiation of fibroblasts into myofibroblasts [5].

Aside from IPF, there are additional diseases of the lungs and other organs that lead to fibrosis. Fibrosis is the result of multiple common steps in such conditions, and PFD targets such pathways. Hence more positive clinical evidence and trials are needed on PFD's usage in different diseases that might improve the quality of life and mortality if used in patients. A random double-blind control study showed PFD to be a promising drug for individuals with overt diabetic nephropathy [6]. Another research indicates that PFD could be useful as an anti-fibrotic in hypertension-induced cardiac hypertrophy [7]. Likewise, PFD has shown an anti-fibrotic effect in thyroid-associated ophthalmopathy [8]. There are other diseases where PFD has been tested, such as chronic hepatitis C and interstitial lung disease associated with systemic sclerosis, and it has shown promising results [9,10].

PFD is a drug that is already approved for IPF. However, much has to be learned about PFD in the real-world population concerning IPF's role in reducing respiratory-related hospitalizations and efficacy in advanced IPF, among few others. Also, only a few studies have explored its use in other fibrotic diseases. In this article, we will review some of the studies about PFD in IPF. Also, we will see PFD's use in other diseases based on the studies that have been done. We intend to encourage scientists to conduct further trials that would help prevent fibrosis progression and, if possible, to reverse it in phenomena where it is known to occur.

## Review

Pirfenidone in idiopathic pulmonary fibrosis 

TGF-β is an important cytokine that facilitates fibrosis. It induces alpha-smooth muscle actin (α-SMA), pro-collagen (Col)-I messenger RNA (mRNA), and protein levels, all of which are attenuated by PFD. Also, TGF-β-induced phosphorylation of Smad3, p38, and a serine/threonine-specific protein kinase (AKT) are inhibited [5]. Additionally, by inhibiting tumor necrosis factor-alpha (TNFα), fibroblast growth factor (FGF), interleukin-one beta (IL-1β), and by reducing platelet-derived growth factor (PDGF) and collagen, type one, alpha one (COL1A1), PFD plays a role in the attenuation of fibroblasts [3,4]. The drug has also exhibited to have either direct or indirect effect on interleukin-six (IL-6), IL-12p40, IL-13, fibronectin, heat shock protein 47 (HSP47), and intercellular adhesion molecule one (ICAM1) [3]. PFD also suppresses the TGF-β-induced fibrotic processes in fibrotic fibroblasts by inversely regulating the collagen triple helix repeat-containing protein one (CTHRC1) [11]. The mechanisms by which the drug PFD acts in IPF seems to be intricate at a molecular level. We will now review some of the clinically observed effects of PFD in IPF.

Most of the patients enrolled in one study on PFD and its effect on respiratory-related hospitalization were men and white. The PFD group had significantly lower respiratory-related hospitalization compared to the placebo. A few reasons for PFD reducing respiratory-related hospitalization could be decreasing acute IPF exacerbation or collateral damage by reducing disease severity. We don't know what kind of respiratory-related hospitalization had been influenced by PFD. Also, it is a post hoc analysis, so data gathered from such analysis should be carefully viewed [12]. Another study showed improved survival in IPF patients admitted to the hospital due to acute exacerbation. These patients were on PFD. The outcome of 11 patients in the PFD group was compared to nine patients in the control group. The sample size was not large [13].

Another study considered many IPF disease progression events such as a relative decline in % forced vital capacity (FVC) > 10%, absolute decline in six-min walk distance >50m, respiratory-related hospitalization, and death. Patients under PFD treatment were less likely to experience multiple disease progression events, and the result was significant compared to placebo. But again, patients were included from the ASCEND and CAPACITY trials; hence most patients were men and mostly white. Also, it was a post hoc analysis [14]. In another study, 170 patients from the same trials were recruited to see PFD's effect on advanced lung dysfunction. They had more advanced lung function impairment. The patients who received PFD had a decreased risk of all-cause mortality and less lung function decline than placebo. However, the sample size was small, and it was a post hoc analysis [15].

An open-label extension study (RECAP) took into account the patients who completed the CAPACITY and ASCEND trials to know PFD's effect on long-term safety. However, there were strict inclusion-exclusion criteria for patient selection in the clinical trials. So, even though the RECAP study findings were consistent with PFD's already known safety profile, they could not be applied to a broader population [16]. In contrast, a retrospective cohort study included patients with many comorbidities and patients with severe IPF disease. It didn't follow any strict inclusion-exclusion criteria like in the phase three trials of IPF. Hence, their findings can be relatively applied to a broader population. The study found an increase in the three-year survival rate in the PFD group, with a 30% survival benefit [17]. Another study determined the long-term safety, efficacy, and feasibility in patients with more advanced IPF. The safety profile and rate of FVC decline in more advanced IPF were similar to less developed IPF. However, the study's drawback was that it had a limited number of patients with more advanced diseases, i.e., 187 for more advanced diseases and 409 for less advanced diseases. It was also a post hoc analysis of the previously discussed open-label extension study (RECAP) in the absence of a placebo arm [18]. Table 1 shows all the studies reviewed for pirfenidone in idiopathic pulmonary fibrosis.

**Table 1:** Pirfenidone in Idiopathic Pulmonary Fibrosis PFD: pirfenidone; IPF: idiopathic pulmonary fibrosis: FVC: forced vital capacity; TGF-β1: transforming growth factor-beta one; CTHRC1: collagen triple helix repeat-containing protein one.

AUTHOR	YEAR OF PUBLICATION	PURPOSE	RESULT/CONCLUSION
Margaritopoulos et al. [17]	2018	To present PFD's effect on survival in patients with IPF along with adverse events in the real world outside clinical trials	The study showed a survival benefit of 30% in the IPF patients compared with the IPF patients treated without the anti-fibrotic and also confirmed the safety profile of PFD
Nathan et al. [14]	2019	To see the effect of more than 12 months of PFD on IPF progression events such as a decline in percent FVC, number of respiratory hospitalizations, decline in 6-min walk distance, and death from any cause	PFD markedly reduced the IPF progression events and death post a progression event
Ley et al. [12]	2017	To see the effect of PFD on nonelective hospitalizations and death after hospitalization in patients with IPF over one year	The effect of PFD in lowering the risk of nonelective respiratory-related hospitalizations in IPF over one year was significant
Costabel et al. [16]	2017	An open-label extension study to gather information about the safety of PFD in long-term use in patients with IPF	The information gathered about the long-term safety of PFD in patients with IPF is consistent with what is known
Costabel et al. [18]	2019	To analyze and know the efficacy and safety of PFD in patients classified under more advanced IPF	After taking PFD, the rate of FVC decline and safety profile in more advanced IPF patients was similar to less advanced IPF patients, and the information suggests that PFD is efficacious, tolerable, and feasible in patients classified under more advanced IPF
Nathan et al. [15]	2019	To assess the safety and efficacy of PFD in patients with advanced IPF	It suggests that PFD is useful in advanced IPF patients without any noticeable increased risk of adverse events
Vianello et al. [13]	2019	To investigate the relationship between PFD and the survival of patients admitted due to acute exacerbation of IPF	Pirfenidone was remarkable in improving the survival of patients that were admitted due to severe acute exacerbation of IPF
Jin et al. [11]	2019	To explore the relationship between lung fibrosis and pirfenidone responses of lung fibroblasts that are induced by TGF-β1	By regulating CTHRC1, whose secretion is induced by TGF-β1, pirfenidone represses fibrosis that is CTHRC1- induced fibroblast mediated. Hence, supporting the use of CTHRC1 in predicting response to pirfenidone
Fraser et al. [4]	2016	To review the management of IPF centered on therapeutic agents	Has provided an overview of the management of IPF and reviewed agents like PFD
Lopez-de la Mora et al. [3]	2015	To shed light on the new findings of PFD about its efficacy	Has called attention to the PFD's effect against inflammation and fibrosis in several diseases that have been studied concerning PFD
Conte et al. [5]	2014	To unfold PFD's targets and the mechanisms of action of the drug	Results have shown PFD's effect in regulating the proliferation of fibroblasts and their differentiation into myofibroblasts by reducing the signaling pathways induced by TGF-β

Pirfenidone in other diseases

Malignant Mesothelioma, Dupuytren's Disease, and Acute Lung Injury

In a study done on PFD in malignant mesothelioma, it was shown that PFD reduced the TGF-β-induced extracellular-signal-regulated kinase (ERK) and serine/threonine-specific protein kinase (AKT) pathways [19]. Not only did PFD reduce the proliferation of cells in mesothelioma, but also it reduced the proliferation of fibroblasts derived from Dupuytren's disease in another study done in vitro [19,20]. The Smad2 phosphorylation induced by TGF-β1 was down-regulated in Dupuytren's disease derived-fibroblasts (DD-fibroblasts); hence PFD acted through the canonical Smad signaling pathway at least partially [20]. While in the study of malignant mesothelioma, the canonical Smad pathway was not affected [19]. PFD inhibited the induction of collagen type one, collagen type three, and fibronectin that happens through TGF-β1 in DD-fibroblasts. Also, the migration and cell contractile properties were reduced by PFD in DD-fibroblasts [20]. In both the studies mentioned above, there was a lot to do with PFD affecting TGF-β. In a study on lung inflammation and fibrosis triggered by acute lung injury done in mice, PFD decreased the lipopolysaccharide (LPS)-induced inflammation and fibrosis [21]. The primary therapeutic target was nod-like receptor (NLR) family pyrin domain containing three (NLRP-3) inflammasome. When the bronchoalveolar fluid was lavaged from the mice given PFD, there was a reduction in the levels of IL-1β and TGF-β1 [21]. Also, in the last-mentioned study here, TGF-β was affected by PFD as well. This tells us the diversity wherein PFD could hold potential usage.

Nonalcoholic Steatohepatitis and Acute Pancreatitis

A study on PFD in non-alcoholic steatohepatitis (NASH) showed that PFD restricted and reversed the steatosis. Diet was used to induce NASH in mice. Many mechanisms were involved through which PFD had its reversing effect. It affected macrophages in a way that T-cell numbers were reduced. PFD upregulated the antioxidant genes, and genes pertaining to the nicotinamide adenine dinucleotide phosphate (NADPH) oxidase complex were downregulated. That way, the oxidant stress was limited. The oxidation of fatty acids was increased, and lipogenesis was decreased. The expression of the inflammatory gene was reduced as well [22]. Another study in mice was done concerning PFD and acute pancreatitis (AP). In that, l-arginine was used to induce pancreatitis. PFD had a protective effect against AP that was induced by l-arginine. The lipid peroxidation was reduced, and antioxidants were intensified [23], a similarity to the previous study mentioned in terms of PFD's effect. Thus, the two studies highlight the antioxidant effect of PFD.

Systemic Sclerosis

Systemic sclerosis affects several organs, and there is fibrosis of the lungs' interstitium as a part of the disease. A multinational study was done on systemic sclerosis-interstitial lung disease (SSc-ILD) wherein patients were recruited from across three countries [24]. Sixty-three patients were involved in the study compared to another study that involved 25 patients with SSc-ILD [24,25]. In the study with 63 patients, there were two groups, one with a two-week titration period and another with a four-week titration period. It was focused on the safety and tolerance of PFD in patients with systemic sclerosis. It was found that adverse events were more common in the two-week titration group than in the four-week titration group. So PFD was more tolerable in the four-week titration group [24]. However, the study doesn't give us much of an idea of the effectiveness of PFD in SSc-ILD. In SSc-ILD, the Hedgehog (Hh) signaling pathway is activated. The other study showed that PFD inhibits the Hh signaling pathway and the glycogen synthase kinase-three beta (GSK‐3β) signaling pathway via the regulation of Sufu expression, focusing on a molecular level [25]. It shows pathways through which PFD could be effective in SSc-ILD. A study concerning PFD and chronic hypersensitive pneumonitis was also done. There was a significant improvement in the change in vital capacity six months after the start of PFD in comparison to the six months before PFD [26]. These findings show that PFD can potentially be used in other conditions outside IPF in the lungs. However, the sample size was small in all three studies, so more studies are needed.

Wound Healing and Existing Scars

Another study researched culturing adult human dermal fibroblasts treated with TGF-β1. Consequently, they differentiated into myofibroblasts. The goal of the study was to test the effect of PFD on differentiated myofibroblasts. This was important because if PFD can act on differentiated myofibroblasts, it could be used on existing scars and for healing wounds. Results were such that the expression of genes that promote fibrosis was down, mainly affecting the expression of alpha-smooth muscle actin. Also, the contractile force was reduced. The findings are promising, but still, it is an in-vitro study [27]. Angiogenesis also plays a role in wound healing and scarring. The study was not focused on that aspect. However, another study focused on PFD in connection with antiangiogenesis. It showed a reduction in the expression of vascular endothelial growth factor levels, through which PFD inhibited various angiogenesis steps in human umbilical vein endothelial cells. Hence, PFD beneficially affected scarring through multiple pathways. This antiangiogenesis effect adds to PFD's anti-fibrotic and anti-inflammatory action against the scarring seen after a glaucoma filtration surgery [28].

Eye Pathologies

Considering PFD's effect on eye pathologies, one study also explored its impact on corneal inflammation post-alkali burn injury in rabbits. PFD lowered IL-β1 expression, an inflammatory cytokine, and TGF-β1, a pro-fibrotic growth factor [29]. Consequently, the corneal haze improved significantly in PFD-treated eyes compared to control eyes. The delivery of PFD was through vitamin-E-loaded contact lenses, which enhanced the bioavailability a lot compared to eye drops. At present, there are many therapies aimed to counter the pathology behind corneal blindness after alkali injury to the eye. A single approach through PFD's anti-inflammatory and anti-fibrotic mechanism seems more logical and promising in this field [29]. There is another study which is about PFD and its effect in preventing proliferative vitreoretinopathy (PVR) in rabbits. PVR is a major consequence of penetrating injury to the globe. There was a significant reduction in the expression of TGFβ, IL-6, and TNFα, as well the collagen-one and alpha-smooth muscle actin (α-SMA) in the PFD treated eyes. When the retina was viewed histologically, there wasn't any significant change in the retina from PFD treated eyes than normal eyes [30]. Both the studies mentioned above concern eyes; injury and blindness may occur in those injuries if not managed. While one pertains to corneal blindness, the other relates to PVR. Both have high inflammatory cytokines and pro-fibrotic growth factors that can be suppressed by PFD, thus preventing the sequelae of blindness. Both are animal studies; hence more studies will be useful.

Transplantation 

A study was done to see the effect of PFD on subsets of T cells with a focus on fighting allograft rejection. Production of cytokines that were associated with Th1 and Th2 was inhibited. Regulatory T cells (Tregs) didn't seem to be affected by PFD. PFD had more impact on CD4+ T cells than CD8+ T cells as observed in vitro; however, in an in vivo model, there was inhibition of both CD4+ and CD8+ cells that was observed. An added effect on the inhibition of CD4+ and CD8+ T cells' proliferation was seen when PFD was used with low-dose rapamycin. The study shows that PFD could potentially be used to fight chronic rejection [31]. A retrospective study was done to see PFD's effect in decreasing lung function decline concerning restrictive allograft syndrome (RAS) post lung transplantation. PFD seemed to lower the decline in the forced vital capacity and forced expiratory volume in one second. However, more extensive studies are needed [32]. Hence, both studies show there is a scope for PFD to be used in the transplantation aspect. Table 2 shows all the studies reviewed for pirfenidone in other diseases.

**Table 2:** Pirfenidone in Other Diseases LPS: lipopolysaccharide; ARDS: acute respiratory distress syndrome; NLRP3: NLR family pyrin containing domain three; GSK‐3β: glycogen synthase kinase-three beta; SSc-ILD: systemic sclerosis-associated interstitial lung disease; TGF-β1: transforming growth factor-beta one; ERK: extracellular signal-regulated kinase; AKT: serine/threonine-specific protein kinase

AUTHOR	YEAR OF PUBLICATION	PURPOSE	RESULT/CONCLUSION
Li et al. [21]	2018	To assess the effect of pirfenidone on LPS-induced inflammation.	Pirfenidone may hold therapeutic potential in ARDS patients by regulating NLRP3 inflammasome activation.
Xiao et al. [25]	2018	To ascertain whether pirfenidone can reduce fibrosis via the hedgehog signaling pathway if used in patients with interstitial lung disease brought about by systemic sclerosis.	By intervening in the hedgehog signaling pathway and the GSK‐3β signaling pathway, pirfenidone has shown an anti-fibrotic effect in interstitial lung disease secondary to systemic sclerosis.
Khanna et al. [24]	2016	To check the tolerability of pirfenidone in patients with systemic sclerosis-associated interstitial lung disease	This study suggests that a longer titration of pirfenidone may be linked to better tolerability even though it shows acceptable tolerability in SSc-ILD patients
Chen et al. [22]	2019	To evaluate pirfenidone as an agent for use in non-alcoholic steatohepatitis in mice	By reducing lipid accumulation and oxidative stress, the study suggests pirfenidone as a potential agent for use in non-alcoholic steatohepatitis.
El-Kashef et al. [23]	2019	To evaluate the role of pirfenidone in acute pancreatitis brought about by I-arginine in mice	Pirfenidone served a protective role in acute pancreatitis brought about by I-arginine in mice
Zhou et al. [20]	2016	To explore the efficacy of pirfenidone with regards to Dupuytren's fibrosis by inhibiting cellular activity mediated by TGF-β_1 _(in vitro)	The fibroblast to myofibroblast transformation was prevented, and extracellular matrix production was inhibited in Dupuytren's disease- derived fibroblasts, suggesting further in-vivo studies with pirfenidone may lead to a new treatment in Dupuytren's disease.
Li et al. [19]	2018	To evaluate the effect of pirfenidone on mesothelioma tumor microenvironment and also on the migration and proliferation of mesothelioma cells	By reducing ERK and AKT pathways as well as the genes associated with the extracellular matrix, pirfenidone not only decreased the migration and proliferation of mesothelioma cells but also altered the mesothelioma tumor microenvironment
Dixon et al. [29]	2018	To test if pirfenidone counters the pathology behind corneal blindness after chemical injury when administered through the contact lens	A marked improvement in corneal haze recognizes pirfenidone as a promising agent to counter corneal inflammation and fibrosis
Khanum et al. [30]	2017	To study the effect of pirfenidone in proliferative vitreoretinopathy secondary to trauma in an animal model	It shows that pirfenidone, when used intravitreally in the setting of ocular trauma, can prevent proliferative vitreoretinopathy.
Liu et al. [28]	2017	An in vitro study to examine the antiangiogenesis effect of pirfenidone	The study shows pirfenidone as a potential multitarget agent against scarring after glaucoma filtration surgery by establishing the antiangiogenesis effect of it in the wound healing process
Wells et al. [27]	2020	An in vitro study to show the effect of pirfenidone on differentiated myofibroblasts	The study indicates that pirfenidone alleviates the effects of differentiated myofibroblasts hence promotes its use in existing scars and healing wounds
Shibata et al. [26]	2018	To assess pirfenidone efficacy in chronic hypersensitivity pneumonitis patients	The study showed remarkable improvement in vital capacity six months after the start of treatment without adverse effects
Vos et al. [32]	2018	To see pirfenidone's effect on lung function in restrictive allograft syndrome post lung transplantation	This study further supports the theory that pirfenidone may reduce the decline in lung function in patients with restrictive allograft syndrome.
Visner et al. [31]	2009	To study the effect of pirfenidone on T-cell function and investigate its immune regulating properties, it can help counter graft rejection.	Pirfenidone could reduce the early transplant response and the fibroproliferative injury, potentially lengthen allograft survival.

Limitations

Animal studies and in-vitro studies were included, among others, in this review. Also, few studies date back more than five years. There were studies discussed under PFD in the IPF section, wherein the population was mainly men and white, and the sample size was small, limiting generalizability. The study population in those studies were from the ASCEND and CAPACITY trials, and the patient selection was through strict inclusion-exclusion criteria. A retrospective study didn't follow those strict inclusion-exclusion criteria; however, the sample size was small. There were no studies as such that could be generalized as per the real-world population.

## Conclusions

Several benefits of PDF's are evident in IPF based on the studies reviewed. It reduced the respiratory-related hospitalization over one year in IPF, improved the survival benefit in patients admitted due to acute exacerbation of IPF, and was shown effective in more advanced IPF, among other benefits. All these studies had a limited sample size, and many of them had their study population from ASCEND and CAPACITY trials (mostly men and white). So, studies on PFD in IPF that can be generalized as per the real-world population are needed. PFD also seems promising in several other diseases where it would provide its anti-fibrotic and anti-inflammatory benefits. It reduced the accumulation and oxidation of lipids in NASH, reduced the proliferation of malignant mesothelioma cells, and inhibited systemic sclerosis pathways that led to fibrosis, among other benefits. Although many studies were preclinical, the results were outstanding. So, PFD has shown effectiveness in many aspects of IPF. Not only that but also it's been useful in other diseases as per the studies. Despite the limitations, the findings of studies tell us that PFD has a vast scope, and future studies in connection with it would change the outcome in many diseases.
